# CircRNA FGFR3 induces epithelial-mesenchymal transition of ovarian cancer by regulating miR-29a-3p/E2F1 axis

**DOI:** 10.18632/aging.103388

**Published:** 2020-07-15

**Authors:** Jing Zhou, Ze-Ning Dong, Bai-Quan Qiu, Ming Hu, Xiao-Qing Liang, Xing Dai, Dan Hong, Yu-Fang Sun

**Affiliations:** 1 Department of Obstetrics and Gynecology, The Forth Affiliated Hospital of Nanchang University, Nanchang 330000, Jiangxi, P.R. China; 2 Xiangya Medical College, Central South University, Hunan 410008, P.R. China; 3 Department of Cardiothoracic Surgery, The Second Affiliated Hospital of Nanchang University, Nanchang 330000, P.R. China

**Keywords:** ovarian cancer, circFGFR3, EMT, E2F1

## Abstract

Circular RNAs (circRNAs) are a class of non-coding RNAs that regulate gene expression after transcription. However, the specific function of circRNAs in ovarian cancer remains undetermined. Previous studies have demonstrated abnormal expression of circFGFR3 in several cancers. The present study was designed to reveal the roles of circFGFR3 in ovarian cancer (OC). CircFGFR3 expression in OC tissues and cells was detected by RT-qPCR. The effects of CircFGFR3 on OC cells were evaluated by transwell assay and CCK-8 assay. Finally, the underlying mechanism was further revealed by luciferase reporter assay and western blotting. Our results showed that circFGFR3 expression was higher in OC cells and tissues than in normal ovarian cells and adjacent normal tissues; in addition, in OC patients, a high level of CircFGFR3 was related to lower survival rates and higher recurrence rates than a low level of circFGFR3. CircFGFR3 overexpression promotes OC progression by inducing epithelial-mesenchymal transition (EMT) in vitro. Mechanistically, circFGFR3 upregulates E2F1 expression by sponging miR-29a-3p, and the overexpression of E2F1 or the suppression of miR-29a-3p induces OC cell EMT. Therefore, circFGFR3 serves as a promoter of OC by inducing OC cell EMT via the miR-29a-3p/E2F1 axis and circFGFR3 may be a prognostic biomarker for OC patients.

## INTRODUCTION

Ovarian malignant tumor is one of the common malignant tumors of female genitalia, second only to cervical cancer and endometrial cancer. Although the incidence of gynecologic malignancies has declined over the years, the mortality rate of this disease remains high and is increasing [1, 2]. Ovarian epithelial cancer has the highest mortality rate among ovarian malignancies and is the deadliest ovarian cancer (OC) among all classifications; it is difficult to diagnose early and treat because early symptoms are not typical [3]. Currently, surgery and chemotherapy combined is still the best treatment for ovarian malignancies [4, 5]. However, the prognosis of patients who undergo surgery and chemotherapy combined is poor (5-year survival rate is low, approximately 30%), and this is closely related to the invasion and metastasis of malignant ovarian cells [6]. Epithelial-mesenchymal transition (EMT) has been revealed to be the primary cause of carcinoma metastasis/invasion [7, 8], including OC [9]. Therefore, it is of great significance to elucidate the underlying molecular mechanism, which may ultimately assist in the advancement of innovative curative strategies against OC.

Previous studies have indicated that non-coding RNAs play an important role in the progression of cancer [10, 11]. CircRNA, one of the non-coding RNA family members, has been identified to be involved in regulating gene expression [12], and the dysregulation of circRNAs has been demonstrated to cause the initiation and progression of cancer. Recently, several studies have reported that the dysregulation of circRNAs occurs in many tumors [13–15]; these studies, showed that circRNAs can act as new biomarkers for predicting the prognosis of patients with cancer. Moreover, a few circRNAs, such as hsa_circ_0061140, circEXOC6B, circN4BP2L2, circHIPK3 and circ-ITCH, have been identified as oncogenes in several cancers, including OC [10, 11, 15, 16]. Therefore, there are still many additional OC-associated circRNAs to be explored.

In the present study, we tried to address the expression and roles of circFGFR3 in OC. We found that circFGFR3 was overexpressed in OC tissues and related to poor prognosis in OC patients. Moreover, we discovered that circFGFR3 upregulated E2F1 expression by sponging oncogenic miR-29a-3p to induce OC cell EMT. Hence, circFGFR3 promotes the OC progression by inducing EMT via the miR-29a-3p/E2F1 axis.

## RESULTS

### circFGFR3 is overexpressed in OC tissues and is associated with poor prognosis in patients

**Figure 1:**
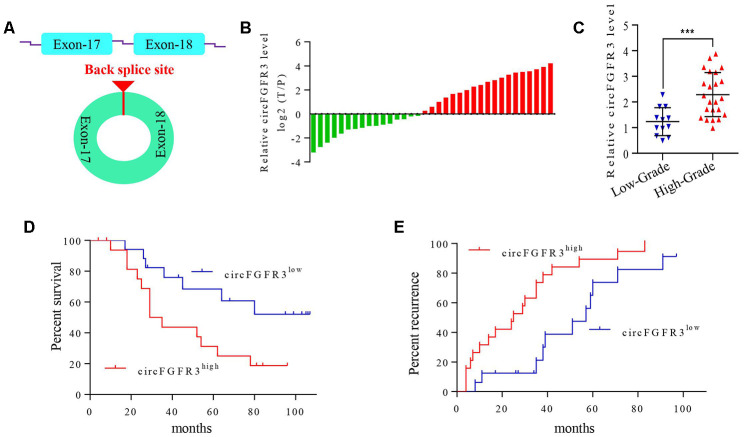
**circFGFR3 is overexpressed in OC tissues and is associated with the patients’ poor prognosis.** (**A**) Schematic illustration showing the circularization of MET exons 17 and 18 forming circFGFR3; (**B**) qRT-PCR were used to detect circFGFR3 expression in OC tissues and adjacent normal tissues, GAPDH served as an internal control. (**C**) The diagram shows the circFGFR3 expression in OC samples from 35 patients divided into stage I-II and stage III-IV groups, ***P<0.005; (**D** and **E**) Kaplan-Meier method was used to analyze the survival and recurrence rates of 35 patients with OC.

First, we detected the level of circFGFR3 derived from exons 17 and 18 of the *FGFR3* gene in 35 paired OC tissues and their adjacent normal tissues by qRT-PCR. Here, total RNA was treated with Ribonuclease R. The results showed that circFGFR3 expression is significantly upregulated in OC tissues (Figure 1A and 1B). Moreover, we found that circFGFR3 expression was closely related to tumor stage, and circFGFR3 expression in stage I-II was significantly lower than that in stage III-IV (Figure 1B). Then, we divided patients into circFGFR3^high^ and circFGFR3^low^ groups. Kaplan-Meier analysis results showed that the circFGFR3^high^ group had a poorer prognosis and higher recurrence rate in OC patients (Figure 1C and 1D). Thus, circFGFR3 expression is higher in OC tissues than in paratumorous tissues, and a high level of circFGFR3 is associated with a worse prognosis in OC patients.

### CircFGFR3 promotes the progression of OC by inducing cell EMT

**Figure 2:**
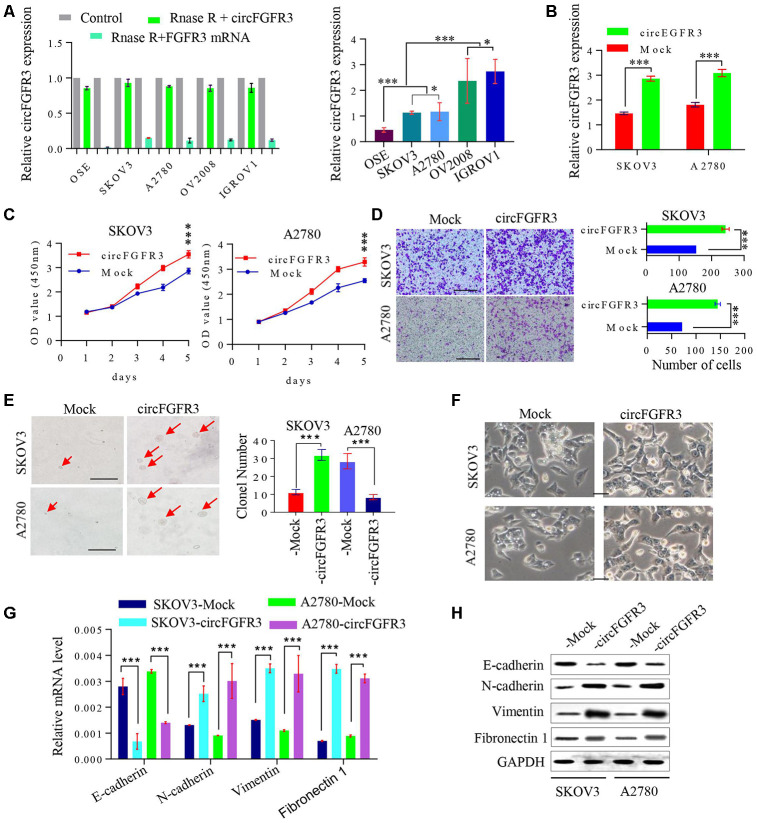
**CircFGFR3 promotes the progression of OC cells.** (**A**) circFGFR3 expression in different OC cell lines was examined by qRT-PCR, GAPDH served as an internal control; (**B**) The expression of circFGFR3 in SKOV3 and A2780 cell lines was upregulated effectively, ***P<0.01; (**C**) Cell viability was determined in circFGFR3-overexpressing OC cells and their control cells, ***P<0.01; (**D**) The invasion of circFGFR3-ovexpressing OC cells and control cells was investigated by Transwell assay, ***P<0.01; (**E**) Clonogenic ability in OC cells with different expression levels of circFGFR3 was determined, Data are shown as the mean ± SD, *** p < 0.001, bar=50 μm; (**F**) The cellular morphology of OC cells with different expression levels of circFGFR3 is shown; (**G** and **H**) Compared to control cell, cells with high levels of circFGFR3 showed a decrease in the epithelial marker E-cadherin, and an increase in the mesenchymal markers vimentin, fibronectin 1 and N-cadherin.

Subsequently, we detected circFGFR3 expression in OC cells after RIP. As shown in Figure. 2A, circFGFR3 expression was lower in SKOV3 and A2780 cells than in OV2008 and IGROV1 cells. Then, we overexpressed circFGFR3 in SKOV3 and A2780 cells by transfecting them with LV- circFGFR3 vectors, and qRT-PCR showed that the circFGFR3 upregulation efficiency was satisfactory (Figure 2B). CCK-8 assays revealed that cell viability was increased after the forced expression of circFGFR3 in A2780 and SKOV3 cells (Figure 2C), and the Transwell assay validated that circFGFR3 upregulation enhanced the invasion of A2780 and SKOV3 cells (Figure 2D). Moreover, we discovered that upregulation of circFGFR3 increased the colony formation of SKOV3 and A2780 cells (Figure 2E). Interestingly, we found that A2780- and SKOV3-Control cells displayed with distinctive cobblestone-like morphology, whereas A2780- and SKOV3-circFGFR3 cells took on a spindle-like morphology (Figure 2F), which indicated that a high level of circFGFR3 might induce OC cell epithelial-mesenchymal transition (EMT). As expected, we detected that E-cadherin was decreased in A2780- and SKOV3-circFGFR3 cells compared to A2780- and SKOV3-Control cells, while, N-cadherin vimentin and Fibronectin 1 (FN1) were significantly increased in A2780- and SKOV3-circFGFR3 cells compared with A2780- and SKOV3-Control cells (Figure 2G and 2H). Together, these data indicate that circFGFR3 overexpression promotes OC development by inducing cell EMT.

### CircFGFR3 functions as a sponge of miR-29a-3p to regulate E2F1 expression in OC cells

**Figure 3:**
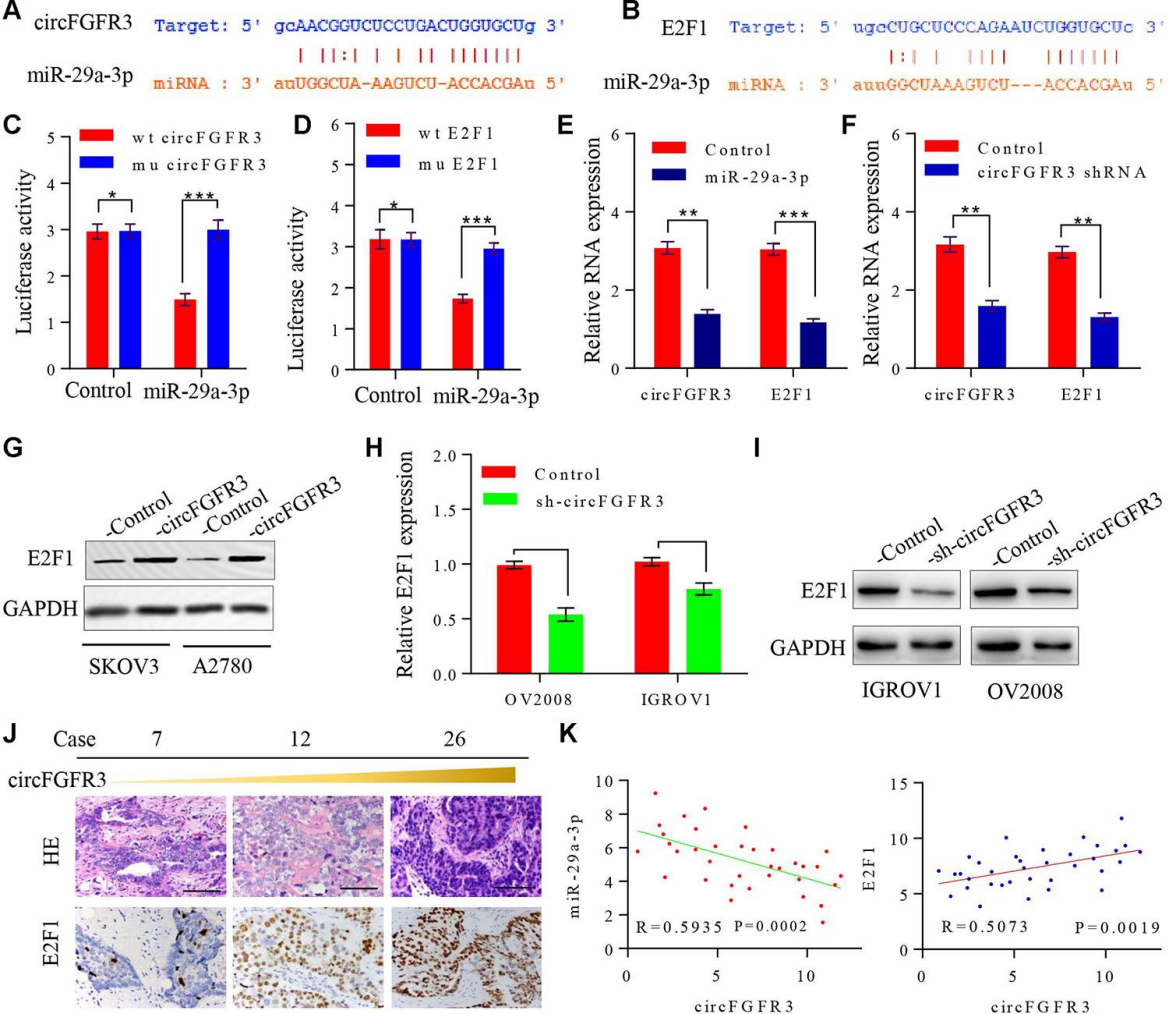
**circFGFR3 as a ceRNA sponge of miR-29a-3p in OC cells.** (**A**) A schematic drawing presenting the putative binding sequence of circFGFR3 and miR-29a-3p; (**B**) A schematic drawing showing the putative binding sequence of miR-29a-3p and the E2F1 3’UTR; (**C** and **D**) Plasmids encoding wild-type and mutated circFGFR3 or E2F1 3’-UTR were transfected into SKOV3 cells with or without miR-29a-3p mimics. Luciferase activity was detected 48 h after transfection. (**E**) circFGFR3 and E2F1 expression changes on the basis of enhanced miR-29a-3p were detected in OC cells by using qRT-PCR. (**F**) circFGFR3 and E2F1 expression changes on the basis of circFGFR3 shRNA expression were detected in OC cells by using qRT-PCR. **P<0.01; ***P<0.05; (**G**) circFGFR3 overexpression increased E2F1 expression in OC cells; (**H** and **I**) circFGFR3 knockdown in OC cells with high levels of circFGFR3 decreased E2F1 expression; (**J**) Representative immunohistochemistry images showing a positive correlation between circFGFR3 and E2F1 in OC tissues, bar=100 μm; (**K**) Diagram showing a negative correlation between miR-29a-3p and circFGFR3 in OC tissues. A positive correlation between circFGFR3 and E2F1 was observed in OC tissues.

A previous study elucidated that circRNAs can sponge miRNAs to prevent their functions in the progression of cancer [17]. Here, we tried to determine the underlying mechanism of circFGFR3 functions in OC cells. First, we identified miR-29a-3p as a target of circFGFR3 with starBase v2.0 (Figure 3A). Furthermore, one binding site for miR-29a-3p in the 3’-UTR of E2F1 was found by bioinformatics analysis (Figure 3B). To verify the effect of circFGFR3 on miR-29a-3p and E2F1, we constructed circFGFR3 and E2F1 pLG3 luciferase reporter plasmids with wild-type and mutated potential binding sites; these studies revealed that miR-29a-3p-overexpressing SKOV3 cells with wild-type circFGFR3 and E2F1 plasmids had lower luciferase activity than control cells, whereas no obvious changes in luciferase activity were observed in SKOV3 cells with mutated binding sites (Figure 3C and 3D). qRT-PCR results revealed that miR-29a-3p mimics suppressed circFGFR3 and E2F1 mRNA expression, while inhibition of circFGFR3 downregulated E2F1 expression in OC cells (Figure 3E and 3F). It has been reported that E2F1 expression is related to the progression of tumors. Then, we hypothesized that circFGFR3 can facilitate the proliferation and migration of OC cells by upregulating E2F1 expression. To elucidate this hypothesis, we performed a western blot assay to analyze changes in the relative E2F1 expression in transfected OC cells. We found that forced expression of circFGFR3 markedly increased E2F1 expression (Figure 3G), while deceased circFGFR3 expression markedly decreased E2F1 expression in OV2008 and IGROV1 cells (Figure 3H and 3I). Importantly, we performed immunohistochemistry to determine E2F1 expression in OC tissues with different expression levels of circFGFR3 and miR-29a-3p, and revealed that circFGFR3 expression was negatively correlated with miR-29a-3p expression and- that there was a positive correlation between circFGFR3 and E2F1 in OC tissues (Figure 3J and 3K). Thus, circFGFR3 can regulate E2F1 expression by affecting the expression of miR-29a-3p.

### Knockdown of E2F1 suppresses circFGFR3-induced OC progression

**Figure 4:**
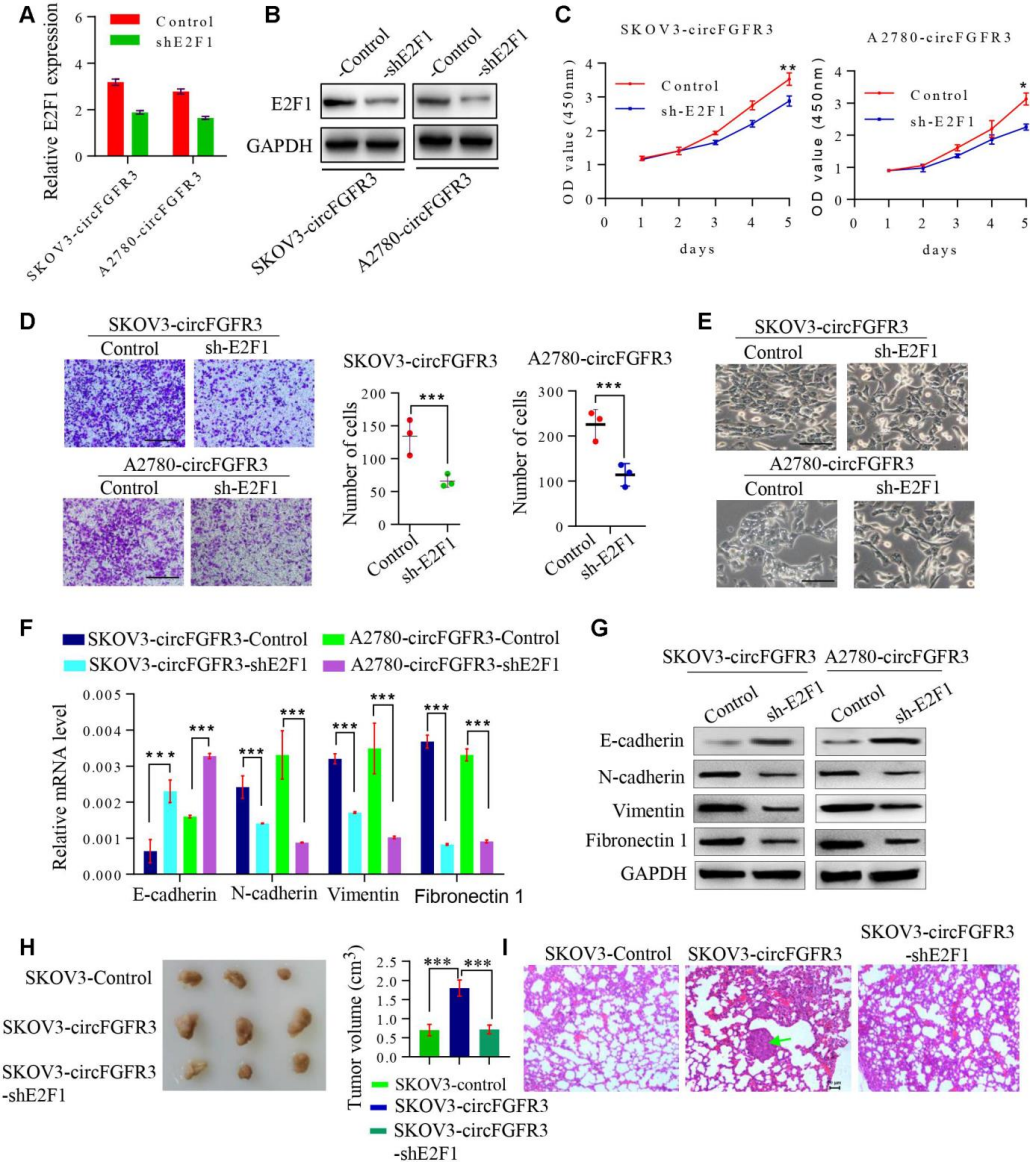
**The knockdown of E2F1 suppresses circFGFR3-induced OC EMT and progression.** (**A**). shRNA interference was used to modify the E2F1 mRNA expression in OC cells. (**B**) Knockdown of E2F1 attenuated circFGFR3-induced upregulation of E2F1 expression in OC cells; (**C**) and (**D**) CCK8 assays were used to measure OC cell viability and Transwell assays were used to measure OC cell migration. Data are represented as the mean ± SD, n=3, *P<0.05; **P<0.01; ***P<0.005, bar=200 μm; (**E**) The cellular morphology of OC cells with circFGFR3 overexpression and different expression levels of E2F1 is shown, bar=200 μm; (**F** and **G**) Cells expressing high levels of circFGFR3 showed an increase in the epithelial marker E-cadherin, and decreases in the mesenchymal markers vimentin, fibronectin 1 and N-cadherin when E2F1 was knocked down. (**H**) Representative images of the tumors from each group (n = 3 mice/group). (**I**) Representative images of the lung to show the metastasis or no metastasis from each group (bar=50nm).

**Figure 5:**
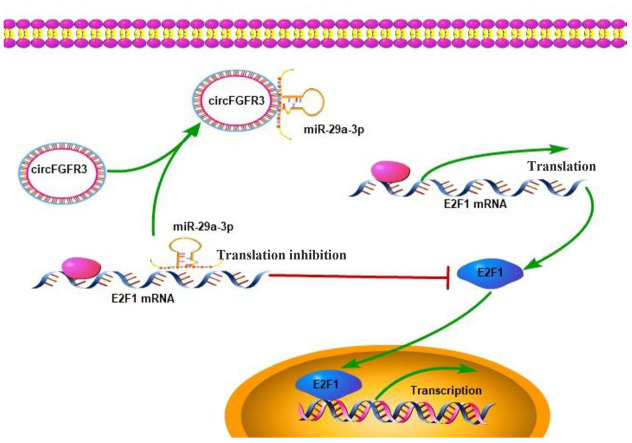
**Working model: An increase in the expression of circFGFR3 promotes the progression of OC.**

CircFGFR3 can enhance E2F1 expression in OC cells. Thus, we attempted to detect whether the oncogenic effect of circFGFR3 can be reversed by interfering with E2F1 expression. First, SKOV3-circFGFR3 and A2780-circFGFR3 cells were transfected with an E2F1 shRNA plasmid and qRT-PCR and western blotting were used to confirm the shRNA knockdown efficacy (Figure 4A and 4B). E2F1 knockdown decreased E2F1 expression and suppressed cell proliferation and migration endowed by circFGFR3 overexpression in vitro (Figure 5C and 4D). Moreover, we further showed that the spindle-like morphology of OC cells expressing high levels of circFGFR3 was also reversed by E2F1 interference (Figure 4E). Commensurately, the epithelial marker, E-cadherin, was upregulated by E2F1 interference in A2780- and SKOV3-circFGFR3 cells, while mesenchymal markers were downregulated by E2F1 interference (Figure 4F and 4G). Additionally, three groups successfully formed tumors (Figure 4H), and the tumor size of SKOV3-circFGFR3-derived xenografts were 1.8 ± 0.21 cm3, which were significantly larger than that of SKOV3-Control (0.70 ± 0.15 cm3) and SKOV3-circFGFR3-shE2F1 (0.72 ± 0.11 cm3) groups. The lung metastasis rates were 100% (3/3) in SKOV3-circFGFR3 group, 0% (0/3) in SKOV3-Control group, and 0% (0/5) in SKOV3-circFGFR3-shE2F1 group (Figure 4I). All the results showed that circFGFR3 might promote OC progression by the miR-29a-3p/E2F1 axis (Figure 5).

## DISCUSSION

It has been reported that non-coding RNAs have become the focus of tumor studies. An increasing numbers of researchers have found that lncRNAs and circRNAs play a vital role in regulating the molecular mechanism of tumors [18]. In our study, we demonstrated that circFGFR3 expression in ovarian cancer tissues was higher than that in adjacent normal ovarian tissues. In OC tissues, the level of circFGFR3 was higher in high-stage OC samples than in low-stage OC samples. Moreover, we found that patients with high circFGFR3 expression had a worse prognosis and a high recurrence rate, which is in line with previous studies in lung cancer, bladder cancer, and cervical cancer [17, 19, 20]. More importantly, we revealed that the high level of circFGFR3 induced OC cell EMT via the miR-29a-3p/E2F1 axis. Given the vital role of EMT in OC demonstrated by previous studies everywhere, we conclude that circFGFR3 overexpression acts as a promoter in OC progression, and can be deemed a reliable marker in predicting prognosis of OC.

Circular RNAs exist universally in many cancers and play many important functions [15, 21–23]. Generally, circRNA functions by sponging miRNAs to compete for endogenous RNAs interactions, thus modulating the stability of mRNAs, regulating gene transcription and translating proteins [24, 25]. Here, we found that overexpression of circFGFR3 in SKOV3 and A2780 cell lines can facilitate cancer cell viability, and migration by inducing cancer cell EMT, which aligns with the previous reports. For example, circFGFR3 has been shown to be overexpressed in non-small cell lung cancer, bladder cancer, pancreatic cancer, and high levels of circFGFR3 promote the development of these tumors [26–29]. Importantly, we demonstrated that circFGFR3 can regulate E2F1 expression by sponging miR-29a-3p. E2F1, a member of the E2F family of transcription factors, has been reported to play a crucial role in the control of the cell cycle [30], as well as induce tumor cell EMT [31]. Additionally, we demonstrated that E2F1 interference reversed the mesenchymal phenotype of cells overexpressing circFGFR3. Thus, the circFGFR3-miR-29a-3p/E2F1 axis is a new pathway in OC progression.

In summary, this study collectively illustrates that circFGFR3 overexpression serves as a major promoter in OC development via the miR-29a-3p/E2F1 axis, and that circFGFR3 has important diagnostic and therapeutic implications for OC patients

## MATERIALS AND METHODS

### Clinical samples

Thirty-five OC and adjacent normal tissues were obtained from patients with ovary serous carcinoma, who underwent complete surgical treatment between January 2011 and December 2011 at the Fourth Affiliated Hospital of Nanchang University (Nanchang, China). In total, the age of patients ranged from 31 to 65 years (mean, 51.6 ± 8. 4 years); 12 patients were classified as low-stage, and 24 patients were classified as high stage. Patients with other malignant tumors were also excluded. Patients with a history of liver-, lung-, kidney- related diseases, or other solid tumors or those who underwent, radiotherapy and chemotherapy were excluded from this study. The histopathological diagnosis of OC was based on the World Health Organization criteria. Suitable formalin-fixed paraffin-embedded tissue samples were subjected to clinicopathological examination, and follow-up data were available for all patients. Ethical consent was accepted by the Committees for the Ethical Review of Research Involving Human Subjects of the Fourth Affiliated Hospital of Nanchang University.

### Cell culture

The human ovarian surface epithelial cell line OSE, and the human ovarian cancer cell lines SKOV3, A2780, OV2008 and IGROV1 were obtained from the Cell Bank of the Chinese Academy of Sciences (Shanghai, China). The culture environment of cells was the same as in a previous study [10].

### Colony formation assay

The colony formation assay was performed as described in previous study [32]. Briefly, 50, 100, and 200 OC cells were cultured in 10-cm dishes. After 2 weeks, the cells were fixed and stained with crystal violet. Finally, representative photographs were captured, the diameter of each cell sphere was determined, and spheres with a diameter greater than 100 μm were grouped as primary spheres.

### Quantitative real-time polymerase chain reaction (qRT-PCR)

Quantitative real-time polymerase chain reaction (qRT-PCR) was performed according to the manufacturer’s instructions. Briefly, we used the TRIZOL kit (Yeasen, Shanghai, China) to extract the total RNAs and used the Reverse Transcription kit (Yeasen, Shanghai, China) to synthesize the cDNA. Then, the SYBR Green PCR kit (Yeasen, Shanghai, China) was used to perform the PCR, and GAPDH served as an internal control [33]. To determine the circRNA in OC and paratumorous tissues, 2 μg total RNA was treated with 3 U/μg Ribonuclease R (BioVision, Inc., Milpitas, USA) for a quarter hour under room temperature. The primers used in this study are listed in Supplementary Table 1. RNA immunoprecipitation (RIP) was carried out according to the manufacturer’s protocol.

### Cell viability assay and Transwell assay

Cell viability was detected by the Cell Counting Kit-8 (CCK-8) (Yeasen, Shanghai, China) and performed according to a previous study [31]. Briefly, cells were inoculated using 96-well plates (1,000 cells/ well). At the indicated times (the 0, 1^st^, 2^nd^, and 3^rd^ days), 10 μl of CCK-8 reagent was added to the wells and incubated for 3 h, the absorbance at 450 nm was detected with Infinite M200 (Tecan, Switzerland). Each sample was assessed

For invasion assays, cells were incubated using 24-well Transwell plates (8 μm pore size, Corning, NY, USA). One million cells suspended in serum-free medium were plated in the upper chambers with Matrigel (BD Biosciences, USA), and 0.6 ml of DMEM or RPMI-1640 medium with 10% FBS was added to the lower chamber. After incubation for a suitable amount of time, cells were fixed in 4% paraformaldehyde, stained with crystal violet and counted under a microscope.

### Transfection

For transfection experiments, miRNA mimics and circFGFR3 overexpression and short hairpin RNA adenovirus were purchased from Genomeditech (Shanghai, China) (Supplementary Table 3). The transfection procedure was performed according to the manufacturer’s instructions, and cells were collected 72 hours after transfection.

### Luciferase reporter assay

Luciferase reporter assays were performed using the Dual-Luciferase Reporter Assay kit (Promega, Madison, WI) as in a previous study [34].

### Western blotting

Cell lysates were collected and centrifuged for 15 min at 12,000 × g and 4 °C. Then, the supernatants- were transferred to clean tubes, and a BCA kit (Pierce, Rockford, IL, USA) was used to quantify the proteins. Western blot assays were performed as described in a previous study [34] and the primary antibodies are included in the Supplementary Table 2.

### Statistical analysis

Statistical analysis was performed with IBM SPSS Statistics 20 (IBM Corp., USA). The values are presented as the means ± standard deviation. Student’s *t*-test was used for comparisons between groups. Correlation analysis was performed between circFGFR3 and miR-29a-3p/E2F1. The recurrence and survival rates were analyzed by the Kaplan-Meier method. *p*<0.05 was considered to be statistically significant.

## Supplementary Material

Supplementary Tables
