# Supplementary material for: CircRNA FGFR3 induces epithelial-mesenchymal transition of ovarian cancer by regulating miR-29a-3p/E2F1 axis
Source: Aging (Albany NY). 2020 Jul 15;12(14):14080–91. doi: 10.18632/aging.103388 (PMC7425466; doi:10.18632/aging.103388)
Supplement: Supplementary Tables [file aging-12-103388-s001..pdf]

SUPPLEMENTARY TABLES

Supplementary Table 1. Sequence of primers for qRT-PCR.

Gene	Forward primer (5'------3')	Reverse primer(5'------3')
circFGFR3	ACACCTGGTTGCTAACCTGGG	CTCCTTCAGCAGCTTGAAGAG
miR-29a-3p	TTCCTCGGTAGCACCATCTG	TATCCTTGTTCACGACTCCTTCAC
E2F1	GCTGGACCACCTGATGAATATCT	GGAGGGGCTTTGATCACCATAA
E-cadherin	CCAAAGCCTCAGGTCATAAACA	AGAAACAGCAAGAGCAGCAGAAT
Vimentin	CCTTGACATTGAGATTGCCACCTA	TCATCGTGATGCTGAGAAGTTTCG
N-cadherin	GTGCCATTAGCCAAGGGAATTCAGC	CGAGGATACTCACCTTGTCCTTGCG
Fibronectin 1	TGCAAGGCCTCAGACCGGGT	GCGCTCAGGCTTGTGGGTGT
GAPDH	TCCACCACCCTGTTGCTGTA	ACCACAGTCCATGCCATCAC

Abbreviations: *q*RT-PCR, quantitative real-time polymerase chain reaction.

Supplementary Table 2. The primary antibodies for western blot.

Antibody	Concentration	Company
E2F1	1/3000	Abcam (Ab179445)
E-cadherin	1:50	Abcam (Ab1416)
Vimentin	1:100	Abcam (Ab92547)
FN1	1:5000	Abcam (Ab207608)
N-cadherin	1/1000	Abcam (ab76057)
GAPDH	1:5000	Abcam(Ab181602)

Supplementary Table 3. shRNA target sequences.

		target sequence/target
sh-circFGFR3-1		
	#1	CCTGGGGCCGGCTGCCCGT
	#2	TGGTTGCTAACCTGGGGCC
sh-E2F1		
	#1	GAAGTCCAAGAACCACATC
	#2	GGAGTCTGTGTGGTGTGTA
	#3	GTCCAAGAACCACATCCAG
